# Bullying behaviours and other conduct problems: longitudinal investigation of their independent associations with risk factors and later outcomes

**DOI:** 10.1007/s00127-021-02062-4

**Published:** 2021-04-15

**Authors:** Keertana Ganesan, Sania Shakoor, Jasmin Wertz, Jessica Agnew-Blais, Lucy Bowes, Sara R. Jaffee, Timothy Matthews, Louise Arseneault

**Affiliations:** 1 Division of Psychology and Language Sciences, University College London, London, UK; 2 Centre for Psychiatry, Wolfson Institute of Preventative Medicine, Queen Mary, University of London, London, UK; 3 Department of Psychology and Neuroscience, Duke University, Durham, NC USA; 4 Social, Genetic, and Developmental Psychiatry Research Centre, Institute of Psychiatry, Psychology, and Neuroscience, King’s College London, London, UK; 5 Department of Experimental Psychology, University of Oxford, Oxford, UK; 6 Department of Psychology, University of Pennsylvania, Philadelphia, PA USA

**Keywords:** Bullying behaviours, Conduct problems, Longitudinal, Predictors, Outcomes

## Abstract

**Purpose:**

Bullying behaviours and other conduct problems often co-occur. However, we do not yet know whether bullying behaviours are associated with early factors and later poor outcomes independently of conduct problems. While there are differing, specific interventions for bullying behaviours and for conduct problems, it is unclear if such specificity is justified given parallels between both behaviours.

**Methods:**

We used prospective data from the Environmental Risk (E-Risk) Longitudinal Twin Study, a nationally representative sample of 2232 children. Mothers and teachers reported on children’s bullying behaviours and conduct problems at ages 7 and 10. We collected measures of risk factors, including temperament and family factors, when children were age 5. We assessed behavioural, emotional, educational and social problems when participants reached the ages of 12 and 18.

**Results:**

Bullying behaviours and conduct problems co-occurred in childhood. Our findings indicated that bullying behaviours and other conduct problems were independently associated with the same risk factors. Furthermore, they were associated with the same poor outcomes at both ages 12 and 18. Despite this, bullying behaviours were uniquely associated with behavioural, emotional, educational and social problems at age 18.

**Conclusions:**

Our findings suggest that anti-bullying programmes and interventions aimed at reducing conduct problems could benefit from greater integration. Furthermore, our study highlights the mental health problems children who bully may face in later years and the need to consider those in intervention plans.

## Introduction

Bullying constitutes a form of repeated, intentional victimisation that commonly takes place between people of the same age group—including peers and siblings—where it is difficult for the victims to defend themselves [1]. Extensive evidence documents harmful outcomes associated with being bullied [2–5]. Findings also show that young people who bully others are at risk of engaging with criminal activities and antisocial behaviours [6]. However, it remains unclear the extent to which bullying behaviours are distinct from other conduct problems in childhood and adolescence and how best to intervene to reduce poor outcomes associated with these often co-occurring behaviours [7, 8].

Bullying behaviour is a criterion for a diagnosis of conduct disorder according to the American Psychiatric Association [9], and bullying could possibly be tackled similarly to other conduct problems. Programmes for reducing conduct problems are typically family-based and focus on parenting skills [10–12]. These programmes typically aim to enhance the knowledge, skills and confidence of parents to manage their children’s behaviour [10]. However, the majority of interventions aimed at reducing bullying behaviours are school-based and focused on changing pupils’ attitudes about bullying through discussions and role playing [6, 13]. There may be a case for augmenting these interventions with elements from interventions aimed at reducing conduct problems. In the present study, we explored the differences and similarities between bullying behaviours and other conduct problems to inform intervention and prevention strategies tackling these prevalent, harmful and costly behaviours [14–16].

Parallels between children who bully others and those with conduct problems can be drawn from epidemiological research. Studies have shown that bullying behaviours and conduct problems are associated with deficits in cognitive abilities such as IQ [17, 18], Theory of Mind [19, 20] and executive functioning [21, 22]. Children who bully others and those with conduct problems are both more likely to have grown up in socioeconomically disadvantaged environments [23–25], and to have antisocial parents [17, 26]. The similarities between these two groups extend to later outcomes. Young people who bully others and those with conduct problems have elevated levels of behavioural and emotional problems [8, 27, 28], delinquency [29], substance use [30–32], difficulties at school [33, 34], and they continue to show violent behaviours in adulthood [35]. Collectively, these findings indicate that bullying behaviours and other conduct problems overlap considerably and might not warrant different intervention approaches.

However, despite considerable similarities, bullying behaviours are arguably distinct from other conduct problems in that they target peers specifically and take place in the context of a power imbalance. The specificity of these behaviours could indicate that peer-related factors (i.e. peer group dynamics) may be particularly relevant for bullying in comparison to other conduct problems [36]; the dyadic relationship between children who bully and their victims could be key for bullying behaviours but not for other conduct problems [37]. Furthermore, bullying behaviours are uniquely associated with callous-unemotional traits, over and above other conduct problems [38]. This finding indicates that bullying behaviours may contribute unique variance to later poor outcomes, further reinforcing that they may be distinct from other conduct problems.

While studies have reported poor outcomes for young people who bully and those with other conduct problems, little research has directly compared to determine the extent to which they are unique. If findings indicate similar risk profiles across these behaviours, then the vast body of evidence that already exists on conduct problems could be used to inform our understanding of bullying behaviours and how to intervene to reduce their prevalence. Furthermore, examining bullying behaviours and other conduct problems in parallel could help ascertain their unique and cumulative contributions to later poor outcomes. Given the frequent co-occurrence of bullying behaviours and conduct problems, it is unclear if bullying behaviours independently predict adverse outcomes later in life, over and above conduct problems. It is possible that associations between bullying behaviours and later outcomes are accounted for by co-occurring conduct problems. Testing this will help address whether there is a need for specific interventions for bullying behaviours, or whether these behaviours could be tackled via existing interventions for conduct problems.

Using data from a UK nationally representative longitudinal cohort, the present study aimed to investigate (i) to what extent childhood bullying behaviours and conduct problems co-occur, (ii) whether established antecedents of conduct problems also predict bullying behaviours, (iii) whether childhood bullying behaviours independently predict behavioural/emotional problems and educational and social difficulties in early adolescence, over and above co-occurring conduct problems, and (iv) whether childhood bullying behaviours independently predict poor outcomes in young adulthood, after taking into account co-occurring conduct problems.

## Methods

### Sample

Participants were members of the Environmental Risk (E-Risk) Longitudinal Twin Study, which tracks the development of a birth cohort of 2232 British children. The sample was drawn from a larger birth register of twins born in England and Wales in 1994 and 1995 [39]. Full details about the sample are reported elsewhere [40]. Briefly, the E-Risk sample was constructed in 1999 and 2000, when 1,116 families (93% of those eligible) with same-sex 5-year-old twins participated in home-visit assessments. This sample comprised 56% monozygotic and 44% dizygotic twin pairs; sex was evenly distributed within zygosity (49% male).

Families were recruited to represent the UK population with newborns in the 1990s to ensure adequate numbers of children in disadvantaged homes and to avoid an excess of twins born to well-educated women using assisted reproduction. The study sample represents the full range of socioeconomic conditions in Great Britain, as reflected in the families’ distribution on a neighbourhood-level socioeconomic index (A Classification of Residential Neighbourhoods, or ACORN, developed by CACI for commercial use) [41, 42].

Follow-up home visits were conducted when the children were 7 years of age (98% participation), 10 years (96%), 12 years (96%), and 18 years (93%). There were 2066 individuals who participated in the E-Risk assessments at age 18. The average age of the participants at the time of the assessment was 18.4 years (*SD* = 0.36); all interviews were conducted after their 18th birthdays. There were no differences between participants who did and did not take part at age 18 in terms of socioeconomic status (SES) assessed when the cohort was initially defined, *χ2* (2, *N* = 2232) = 0.86, *p* = 0.65; age-5 IQ scores, *t* (2208) = 0.98, *p* = 0.33; or age-5 emotional or behavioural problems, *t* (2230) = 0.40, *p* = 0.69, and *t* (2230) = 0.41, *p* = 0.68, respectively.

Home visits at ages 5, 7, 10, and 12 years included assessments with participants as well as their mother (or primary caretaker). Teachers’ reports were collected via postal questionnaires (posted to the children’s teachers, with parents’ permission). The home visit at age 18 included interviews only with the participants. The joint South London and Maudsley–Institute of Psychiatry, Psychology & Neuroscience Ethics Committee approved each phase of the study. Parents gave informed consent and twins gave assent between 5 and 12 years and then informed consent at age 18 years.

### Measures

#### Bullying behaviours and other conduct problems

We assessed bullying behaviours using mothers’ and teachers’ reports when participants were ages 7 and 10 with items from the Children’s Behavior Checklist [43] and Teacher’s Report Form [44] (‘bullying or threatening people’, ‘cruel or nasty to other people’, and ‘teases a lot’ and teachers’ report for the items ‘cruelty, bullying, or meanness to others’, ‘teases a lot’, and ‘threatens people’). Mothers and teachers were asked to rate each item as being ‘not true’ (0), ‘somewhat or sometimes true’ (1), or ‘very or often true’ (2). The internal consistency for the combined mother and teacher ratings was 0.66 at age 7 and 0.69 at 10.

We assessed conduct problems—other than bullying behaviours—at ages 7 and 10 using items from the Delinquent Behavior (e.g., ‘lying or cheating’) and Aggressive Behavior scales (e.g., ‘temper tantrums or hot temper’) of the Child Behavior Checklist [43] and Teacher’s Report Form [44], supplemented with DSM–IV items assessing conduct disorder (e.g., ‘stays out at night past the time he/she should be home’). The internal consistency reliabilities for combined ratings from mothers and teachers were 0.93 at age 7 and 0.94 at age 10.

Scores were averaged across informant and time to create a summary measure capturing pervasive and persistent bullying behaviours and other conduct problems. Combining mother and teacher ratings allowed us to capture behaviours in different settings (i.e. school and home environments). Inter-rater reliability estimates were comparable between the bullying behaviour scales (age-7 mothers–teachers *r* = 0.23; age-10 mothers–teachers *r* = 0.25) and the conduct problem scales (age-7 mothers–teachers *r* = 0.30; age-10 mothers–teachers *r* = 0.30).

#### Risk factors and outcomes of bullying and other conduct problems

We selected possible predictors and outcomes of bullying behaviours and other conduct problems based on previous research. Details are reported in Table 1.

**Table 1:** Measures of bullying behaviours, conduct problems, antecedents and outcomes

	Measure	Informant	Mean (SD) or %	Observed range	Inter-rater reliability (*r*)	Reference citations
Bullying and other conduct problems						
Bullying behaviours	Child Behavior Checklist (CBCL), Teacher Report Form (TRF)	Mother, Teacher	0.61 (0.74)	0–5	0.66–0.69	[69]
Other conduct problems	Child Behavior Checklist (CBCL), Teacher Report Form (TRF), DSM-IV Items	Mother, Teacher	0.86 (1.21)	0–8.5	0.93–0.94	[43]
Age-5 predictors						
Undercontrolled temperament	Children’s approach and response to interview	Interviewer	2.41 (3.63)	0–18	–	[70]
Child maltreatment	Adapted parenting interview schedule	Mother	14	0–1	0.9	[71, 72]
Low maternal warmth	Maternal expressed emotion scale based on the 5-min speech sample method	Rater coded	3.30 (1.00)	0–5	0.9	[73]
Domestic violence	Conflicts Tactics Scale and 3 items assessing other abusive behaviours	Mother	42	0–1	–	[74]
Parents’ antisocial behaviour	Young adult behaviour checklist supplemented with questions from Diagnostic Interview Schedule (DIS) for DSM-IV	Mother	27.58	0–1	–	[75, 76]
Low socio-economic status	Standardized composite of income, education and social class modelled on the British Social Attitudes survey	Mother	33.24	0–1	–	[77]
Age-12 outcomes						
Antisocial behaviour	Computer task based on DSM	Self-report	2.46 (2.94)	0–24	–	[78]
Substance use	Computer based questionnaire	Self-report	0.21 (0.53)	0–5	–	–
Depression symptoms	Children’s Depression Inventory (CDI)	Self-report	3.11 (5.32)	0–42	–	[79]
Anxiety symptoms	Multidimensional Anxiety Scale for Children (MASC) with interview	Self-report	7.62 (3.04)	0–18	–	[80]
Academic difficulties	Computer based questionnaire	Self-report	0.34 (0.60)	0–2	–	–
Low popularity	Computer based questionnaire	Self-report	26.57	0–1	–	–
Age-18 outcomes						
Antisocial behaviour	Interview based on Diagnostic and Statistical Manual of Mental Disorders (DSM-IV) criteria	Self-report	2.12 (2.28)	0–11	–	[76]
Criminal behaviour	UK Police National Computer (PNC) record searches	Official record	10.78	0–1	–	–
Alcohol dependence symptoms	Interview based on Diagnostic and Statistical Manual of Mental Disorders (DSM-IV) criteria	Self-report	1.13 (1.67)	0–11	–	[76]
Cannabis dependence symptoms	Interview based on Diagnostic and Statistical Manual of Mental Disorders (DSM-IV) criteria	Self-report	0.24 (0.98)	0–7	–	[76]
Depression symptoms	Interview based on Diagnostic and Statistical Manual of Mental Disorders (DSM-IV) criteria	Self-report	1.81 (2.97)	0–9	–	[76]
Anxiety symptoms	Interview based on Diagnostic and Statistical Manual of Mental Disorders (DSM-IV) criteria	Self-report	0.95 (1.82)	0–6	–	[76]
Academic difficulties	Not in Education, Employment, or Training (NEET) interview	Self-report	11.57	0–1	–	[81]
Social isolation	Scale of Perceived Social Support (MSPSS)	Self-report	3.29 (4.34)	0–24	0.88	[82]

### Statistical analyses

First, we calculated correlations to examine the extent to which bullying behaviours and conduct problems co-occurred at ages 7 and 10. Second, we used linear regression models to test whether childhood risk factors were associated with bullying behaviours and conduct problems. More specifically, we examined whether risk factors predicted bullying behaviours and conduct problems individually in bivariate models. Furthermore, we examined whether these risk factors predicted bullying behaviours and other conduct problems after controlling for each other. Third, using linear and logistic regression models, we tested whether bullying behaviours and conduct problems at ages 7 and 10 were similarly associated with later difficulties at ages 12 and 18. Initially, we tested whether each outcome was associated with bullying behaviours and conduct problems separately in bivariate models. To test the unique contributions of childhood bullying behaviours, we tested whether each outcome was associated with bullying in multivariate models controlling for concurrent childhood conduct problems. The same strategy was employed to examine the unique contribution of childhood conduct problems where concurrent childhood bullying behaviours were controlled for.

We used moderation analyses to check whether the associations differed by sex. Regression analyses with sex-interaction terms did not yield significant improvements in the fit of models above and beyond models with main effects only. Thus, analyses conducted for the whole sample were collapsed across sex. We used the Huber–White or Sandwich estimator [45] to obtain robust standard errors, to account for the non-independence of twin data. All analyses were conducted using Stata 12.0 [46].

## Results

### To what extent do bullying behaviours and other conduct problems co-occur in childhood?

**Fig. 1:**
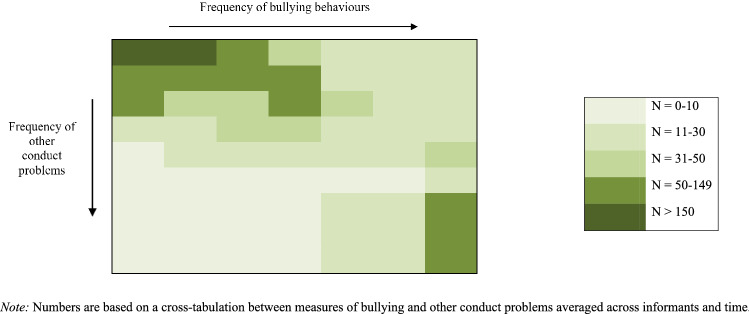
Overlap between bullying behaviours and other conduct problems

Children’s bullying behaviours at ages 7 and 10 went hand-in-hand with other conduct problems. Bullying behaviours and conduct problems were significantly correlated at age 7 (*r* = 0.62, *p* < 0.001) and age 10 (*r* = 0.66, *p* < 0.001). In addition to this, bullying behaviours and conduct problems were significantly correlated across time points (*r* > 0.4, *p* < 0.001). Figure 1 illustrates that very few participants showed frequent bullying behaviours in the absence of other conduct problems, and vice versa.

### Are age-5 risk factors associated with childhood bullying behaviours and other conduct problems?

Children who at age 5 had an undercontrolled temperament, had been exposed to low maternal warmth, maltreatment or domestic violence, had parents with antisocial behaviour, or who had experienced socioeconomic disadvantage showed more frequent bullying behaviours and conduct problems compared to children who were not exposed to these risk factors (Table 2). Associations with these risk factors and bullying behaviours reduced after accounting for concurrent conduct problems; only low maternal warmth remained independently associated with bullying behaviours, indicating that this risk factor is specifically associated with children’s bullying behaviours, independent of other conduct problems. Undercontrolled temperament, low maternal warmth and parents’ antisocial behaviour also predicted conduct problems after adjustment for co-occurring bullying behaviours.

**Table 2:** Models predicting bullying behaviours from children’s early adversity and co-occurring conduct problems from bivariate and multivariate analysis

	Age-7 and 10 bullying behaviours and conduct problems			
	Bullying behaviours	Conduct problems	Bullying behaviours controlling for other conduct problems	Conduct problems controlling for bullying behaviours
Age-5 predictors	β (95% CI)	β (95% CI)	β (95% CI)	β (95% CI)
Undercontrolled temperament	**0.15 (0.09, 0.21)**	**0.16 (0.10, 0.23)**	**0.04 (0.00, 0.07)**	**0.06 (0.02, 0.10)**
Child maltreatment	**0.16 (0.10, 0.21)**	**0.13 (0.07, 0.19)**	**0.07 (0.03, 0.10)**	0.02 (− 0.02, 0.06)
Low warmth	**0.09 (0.02, 0.16)**	**0.13 (0.05, 0.20)**	0.00 (− 0.04, 0.04)	**0.06 (0.02, 0.11)**
Domestic violence	**0.20 (0.15, 0.26)**	**0.16 (0.11, 0.22)**	**0.09 (0.05, 0.12)**	0.02 (− 0.02, 0.06)
Parents’ antisocial behaviour	**0.23 (0.17, 0.29)**	**0.22 (0.15, 0.28)**	**0.08 (0.05, 0.12)**	**0.06 (0.02, 0.09)**
Low socioeconomic status	**0.20 (0.15, 0.26)**	**0.17 (0.11, 0.23)**	**0.09 (0.05, 0.12)**	0.03 (− 0.01, 0.07)

Significant associations have been shown in bold

Associations are expressed as standardised regression coefficients (β) with 95% Confidence Interval (CI). Residuals of regression analysis with bullying behaviours and conduct problem were normally distributed. Log-transformation of variables did not affect the observed associations

### Are bullying behaviours and other conduct problems in childhood independently associated with poor outcomes at age 12?

Frequent bullying behaviours and other conduct problems predicted worse outcomes at age 12. Bullying behaviours and conduct problems were both associated with higher levels of behavioural problems later on and increased symptoms of depression (Table 3). Associations with anxiety did not reach statistical significance. Only conduct problems were associated with more educational and social problems. After adjusting for conduct problems, bullying behaviours were no longer significantly associated with later depression. Additionally, effect sizes for antisocial behaviour and substance use were attenuated by between 37 and 39%, but remained statistically significant. In contrast, after adjusting for bullying behaviours, conduct problems remained significantly associated with all outcomes, with associations attenuated by between 5 and 45%.

**Table 3:** Models predicting age-12 outcomes with childhood bullying behaviours and conduct problems

	Age-12 outcomes					
	Behavioural problems		Emotional problems		Educational and social difficulties	
Age 7/10	Antisocial behaviour	Substance use	Depression symptoms	Anxiety symptoms	Academic difficulties	Low popularity
	β (95% CI)	β (95% CI)	β (95% CI)	β (95% CI)	β (95% CI)	OR (95% CI)
Unadjusted for co-occurrence						
Bullying behaviours	**0.30 (0.24, 0.36)**	**0.18 (0.12, 0.24)**	**0.14 (0.07, 0.20)**	0.01 (− 0.04, 0.06)	**0.13 (0.07, 0.20)**	1.10 (0.99, 1.23)
Conduct problems	**0.29 (0.23, 0.36)**	**0.18 (0.12, 0.24)**	**0.21 (0.13, 0.28)**	0.04 (− 0.02, 0.10)	**0.17 (0.10, 0.25)**	**1.22 (1.10, 1.35)**
Adjusted for co-occurring problems at ages 7 and 10						
Bullying behaviours	**0.19 (0.11, 0.26)**	**0.11 (0.04, 0.19)**	− 0.02 (− 0.08, 0.05)	− 0.03 (− 0.10, 0.03)	0.02 (− 0.05, 0.08)	0.90 (0.78, 1.07)
Conduct problems	**0.16 (0.09, 0.23)**	**0.10 (0.02, 0.18)**	**0.22 (0.13, 0.31)**	0.06 (− 0.01, 0.13)	**0.16 (0.08, 0.25)**	**1.30 (1.12, 1.51)**

OR (95% CI)—odds ratio with 95% confidence interval. β (95% CI)—beta coefficient value with 95% confidence interval

Significant associations have been shown in bold

### Are bullying behaviours and other conduct problems in childhood independently associated with poor outcomes at age 18?

Similar to age-12 outcomes, we observed that frequent bullying behaviours and other conduct problems were associated with poor outcomes at age 18. Bullying behaviours and conduct problems were associated with antisocial and criminal behaviours, symptoms of alcohol and cannabis dependence, symptoms of depression, and educational and social difficulties (Table 4). Once more, we did not find statistically significant associations with symptoms of anxiety. After adjusting for conduct problems at age 12, associations with bullying behaviours and age-18 outcomes remained moderate and statistically significant (though attenuated up to 31%). After adjusting for bullying behaviours, associations between other conduct problems and symptoms of alcohol and cannabis dependence, depression and academic difficulties became non-significant. Associations between conduct problems with antisocial behaviour, criminal behaviour and social isolation remained significant (though reduced by between 7 and 52%).

**Table 4:** Models predicting age-18 outcomes with childhood bullying behaviours and conduct problems

	Age-18 outcomes								
		Behavioural problems				Emotional problems		Educational and social difficulties	
Age 7/10		Antisocial behaviour	Criminal behaviour	Alcohol dependence symptoms	Cannabis dependence symptoms	Depression symptoms	Anxiety symptoms	Academic difficulties	Social isolation
		β (95% CI)	OR (95% CI)	β (95% CI)	β (95% CI)	β (95% CI)	β (95% CI)	OR (95% CI)	β (95% CI)
Unadjusted									
Bullying behaviours		**0.26 (0.20, 0.32)**	**1.94 (1.70, 2.22)**	**0.12 (0.07, 0.17)**	**0.17 (0.09, 0.25)**	**0.09 (0.05, 0.14)**	0.03 (− 0.02, 0.08)	**1.60 (1.42, 1.80)**	**0.12 (0.06, 0.17)**
Conduct problems		**0.25 (0.19, 0.31)**	**1.81 (1.59, 2.07)**	**0.09 (0.04, 0.14)**	**0.14 (0.05, 0.23)**	**0.08 (0.03, 0.12)**	0.03 (− 0.02, 0.08)	**1.49 (1.32, 1.68)**	**0.15 (0.09, 0.21)**
Adjusted for co-occurring problems at ages 7 and 10									
Bullying behaviours		**0.18 (0.11, 0.25)**	**1.61 (1.33, 1.96)**	**0.12 (0.04, 0.19)**	**0.14 (0.07, 0.22)**	**0.08 (0.02, 0.15)**	0.02 (− 0.05, 0.08)	**1.48 (1.24, 1.76)**	0.02 (− 0.05, 0.09)
Conduct problems		**0.12 (0.04, 0.19)**	**1.28 (1.06, 1.56)**	0.00 (− 0.07, 0.07)	0.04 (− 0.05, 0.13)	0.02 (− 0.05, 0.08)	0.02 (− 0.05, 0.08)	1.11 (0.93, 1.34)	**0.14 (0.06, 0.22)**

*Note:* OR (95% CI)—odds ratio with 95% confidence interval. β (95% CI)—beta coefficient value with 95% confidence interval

Significant associations have been shown in bold

## Discussion

Who are those children who bully others and what can we do to tackle these behaviours? Findings from our nationally representative longitudinal cohort of British children shed light on these questions and provide new insight uncovering whether children who bully others and those with conduct problems are distinct from one another. First, we showed that bullying behaviours do not occur in isolation and are most often accompanied by other conduct problems. Second, well-established risk factors for childhood conduct problems are also associated with bullying behaviours, independently of other conduct problems. Third, despite the overlap between both types of behaviours and their shared predictors, they independently predict poor outcomes in later life. This suggests that bullying behaviours and other conduct problems may be better addressed by multi-level interventions that include parents, teachers, and peers.

Simultaneously examining bullying behaviours and other conduct problems allowed us to compare and contrast the profiles of both behaviours and examine their specificity. Our study provides evidence that children who bully others and those with conduct problems share many characteristics. This builds on prior research that looked at these groups separately [1, 23–26, 47]. Children who bully and those with other conduct problems were both at increased risk of developing poor outcomes in early adolescence and young adulthood, independently of each other. Our findings highlight that bullying behaviours may foreshadow antisocial and criminal behaviours in later life, in a similar manner to other conduct problems [48]. In addition to showing continuity over time, both types of behaviours were also associated with later depression, as well as educational and social problems, but not with anxiety. These findings highlight that children who bully develop behavioural, emotional, educational and social problems, similar to children with other conduct problems [33, 49].

Despite showing similarities between the two types of behaviours, our findings indicate that bullying behaviours uniquely contributed to later poor outcomes. In early adolescence, bullying behaviours were independently associated with antisocial behaviour, substance use and low popularity. In young adulthood, they were independently associated with all types of behavioural problems, and also depression and academic difficulties. The association with depression may potentially be explained by the social nature of bullying behaviours. Because bullying takes place between peers, it may impinge on the likelihood of establishing positive peer relationships which are important sources of support for young people [50, 51]. Social support has been found to buffer against mental health problems in times of stress [52]. Indeed, studies have found that those who have a stronger social support network and high-quality friendships have lower emotional and behavioural problems than children without [53, 54]. While some studies reported that young people who bully were considered popular by their peers [55–57], this may not reflect positive and supportive relationships that are needed to reduce the risk of mental health problems. The longitudinal association with depression expands upon previous literature showing that behavioural problems in early childhood predict emotional problems in mid-childhood [8, 23, 49, 58], with one meta-analysis showing that childhood bullying specifically contributes to later depression [59]. Our findings are consistent with a ‘failure’ model [60], which proposes that youth with behavioural problems develop emotional problems as they grow older because of the negative experiences they have encountered as they grow up including academic failures and poor family and peer relationships. These findings highlight the detrimental nature of childhood bullying and its unique contribution to later poor outcomes extending into young adulthood.

This brings us to discuss the limits of our study. First, we did not have any measures that would have allowed us to examine peer factors that may be centric to the uniqueness of bullying behaviours. Examining peer dynamics and interpersonal functioning with peers could further clarify why bullying behaviours uniquely contribute to later problems. Second, we did not use a standardised instrument specific to assessing bullying behaviours. Rather we extracted items relevant to bullying behaviours from an instrument used to assess a variety of problem behaviours more broadly. Nevertheless, we identified antecedents and later outcomes amongst children who bully similar to those shown in previous studies that used standardized bullying measures [8, 29, 35, 61], suggesting construct validity. Furthermore, we used both mothers’ and teachers’ reports to measure bullying and conduct problems, which may capture behaviours observed in different settings [62] and reduce concerns related to shared method variance. Second, when investigating the similarities between children who bully and children with other conduct problems, we did not distinguish between the types of bullying and conduct problems. This would have allowed us to further investigate the underlying mechanisms that contribute towards the similarities and differences between children who bully and children with other conduct behaviours. Third, young adult outcomes were restricted to age 18, and therefore long-term outcomes were not captured with these data. However, age 18 is a critical period for the developmental trajectory of antisocial behaviours [63–66], and thus behaviours measured at this time point may be key to capture salient poor outcomes. Fourth, we restricted the analyses to examining bullying behaviours and conduct problems in childhood only. We did not examine how later bullying and conduct problems in mid-childhood may uniquely contribute to problems in later years. Therefore, our findings are specific to outcomes of earlier bullying and conduct problems. The onset of bullying behaviours in adolescence may potentially have a varied unique contribution to later problems which future research is required to examine. Fifth, as our study includes twin pairs, it is unclear if results are generalisable to the population. However, previous studies have found the rates of psychopathology in singletons and twins are comparable [67]. Sixth, we did not test if controlling for earlier risk factors mitigates the associations observed between bullying behaviours and later poor outcomes. It is possible that these risk factors account for any observed associations between bullying behaviours and poor outcomes. However, this does not take away from the take home message that bullying behaviours independently increase the risk for emotional and behavioural problems later on.

Our findings have implications for future research and interventions. Despite the overlap between bullying behaviours and conduct problems, our findings suggest there is value in examining bullying behaviours specifically as they are associated with worse outcomes later on. In addition, our findings demonstrate the importance of controlling for conduct problems when investigating the outcomes associated with bullying behaviours. Some associations between bullying behaviours and poor outcomes became non-significant after adjusting for co-occurring conduct problems. This highlights the risk of spurious correlations when conduct problems are not accounted for. Finally, our findings may help inform interventions targeting bullying behaviours. Our study suggests that interventions aimed at preventing bullying behaviours could be combined with those tackling conduct problems, given similar sets of risks factors for both types of behaviours. Specifically, like conduct problems, bullying behaviours were associated with risk factors within the family environment. This suggests that although bullying is often regarded as schools’ responsibility to tackle, our findings suggest that it is necessary for interventions targeting bullying behaviours to include a family component, rather than being exclusively school-based. In particular, Fast Track targeting conduct problems is multisite, targeting behaviours both at home and at school [68]. Bullying behaviours may benefit from such multisite interventions, addressing familial factors that may contribute to bullying behaviours alongside targeting bullying behaviours at school. Moreover, our findings emphasise that we need to acknowledge children who bully others may also experience emotional problems. Interventions should not only focus on curbing their antisocial behaviours but should also consider their risk of facing later depression and other educational and social problems.

In conclusion, the present study showed that bullying behaviours frequently co-occur and share risk factors with other conduct problems, suggesting that interventions aiming to prevent these behaviours could be combined. However, our findings also showed that these behaviours uniquely contribute to poor outcomes both in mid-childhood and adulthood. Thus, programmes aiming to reduce poor outcomes among children showing these types of behaviours should regard bullying behaviours distinctly and consider mental health needs for children who bully others.
